# Impact of Respiration on LV Volume and Function Using rt-MRI

**DOI:** 10.1186/1532-429X-18-S1-P329

**Published:** 2016-01-27

**Authors:** Francisco Contijoch, Sebastian Berisha, Joseph H Gorman, Robert C Gorman, Walter R Witschey, Yuchi Han

**Affiliations:** grid.25879.310000000419368972University of Pennsylvania, Philadelphia, PA USA

## Background

ECG-gated cardiac MRI acquired during breathholds is the gold standard for volumetric evaluation of patients, and clinically, ejection fraction is used as a surrogate for function. We hypothesized that the breathholds alter hemodynamic measurements by changing the loading conditions of the heart as well as the heart rate. Real-time MRI and semi-automated LV endocardial segmentation can be used to quantify slice volume during respiration. We derive global hemodynamic measurements during breathholds and free respiration to measure changes related to respiration.

## Methods

Short-axis golden angle radial bSSFP projections (8000 - 12000 projections/slice) were reconstructed using Gadgetron (non-Cartesian, iterative SENSE) with 34 projections per frame, and slice volume was measured via segmentation of LV endocardial contour with ITK-SNAP. A respiratory navigator was obtained by projection through the diaphragm and respiratory phases were identified. For breathheld acquisitions, a single hemodynamic measurement was obtained. For free breathing scans, global hemodynamics were calculated for end-inspiratory and end-expiratory periods.

## Results

7 clinical patients were imaged as part of ongoing cardiac MRI research studies. The LV EDVs and LV EFs acquired during breathheld acquisitions were not statistically different from selection of end-expiration during free breathing acquisition (p = 0.26 and p = 0.84, respectively). However, LV EDV at end-inspiration was 9.00 ± 7.21 mL (p = 0.02) higher and LV EF was 3.92 ± 1.62 % (p < 0.01) lower compared to values obtained from breathheld data. Finally, we compared the LV EDV and LV EF values obtained at the two respiratory phases within the free breathing acquisition. The end-expiration EDV was not significantly different but the end-expiration EF was 3.91 ± 1.52 % higher than end-inspiration (p < 0.01).

## Conclusions

Breathheld acquisitions during cine MRI eliminate respiratory motion, but are unable to observe the respiratory effect on hemodynamic function of a patient. Using real-time MRI during free breathing, we have observed the natural variation in LV EDV and EF and found comparable values within end-expiratory periods when compared to standard breathholding.

**Figure 1:**
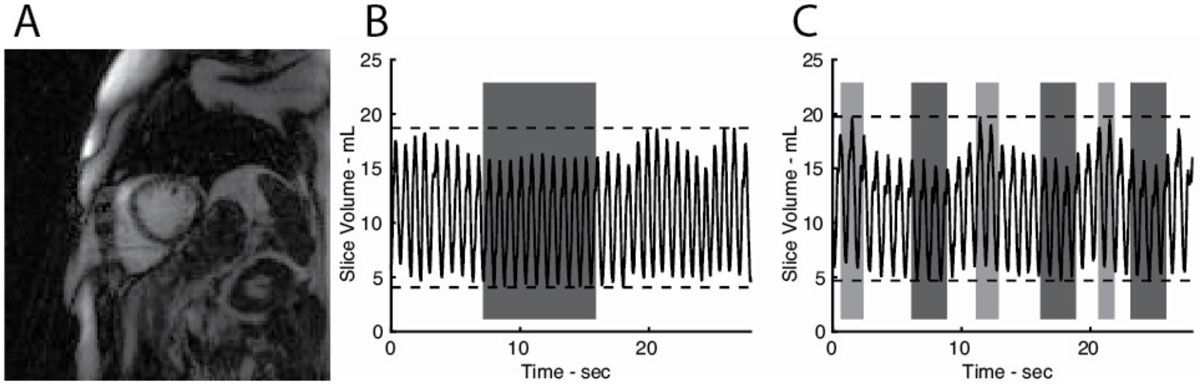
**Effect of Respiratory Motion on MRI-derived Hemodynamic Measures**. **A** Example of RT MRI Image in Short-Axis Slice. **B** Slice volume measured during a conventional breathhold. Gray box indicates period selected for hemodynamic measurements during breathhold. **C** Slice volume measured during free breathing acquisition. End-inspiration and end-expiration periods are demarcated with light and dark gray boxes, respectively.

